# Racial differences in central adiposity in a longitudinal cohort of black and white adolescent females

**DOI:** 10.1186/1471-2431-10-2

**Published:** 2010-01-21

**Authors:** David J Tybor, Alice H Lichtenstein, Gerard E Dallal, Stephen R Daniels, Aviva Must

**Affiliations:** 1 Friedman School of Nutrition Science and Policy at Tufts University, Boston, MA, USA; 2 Jean Mayer USDA HNRC at Tufts University, Boston, MA, USA; 3 University of Colorado School of Medicine, Denver and Aurora, Colorado, USA; 4 Tufts University School of Medicine, Boston, MA, USA

## Abstract

**Background:**

Central adiposity is related to chronic disease risk in adolescents. Racial differences in waist circumference have been identified using cross-sectional data from this age group. We tested for racial differences in age-related growth in waist circumference in a longitudinal cohort of black and white adolescent girls.

**Methods:**

We analyzed 9 years of publicly available data from the National Heart, Lung, and Blood Institute Growth and Health Study, for 2379 girls (1213 black and 1166 white) enrolled at age 9-10 years in 1987-1988 and followed annually. Individual growth trajectories of waist circumference were constructed for girls with >3 annual measures. Mixed models were used to compare changes in waist circumference during adolescence between black and white females. BMI and age at menarche were included in the models.

**Results:**

At each age, black females had significantly higher waist circumference. Mean annual increase in waist circumference was significantly higher for black females compared to white females (1.46 cm/yr vs. 1.36 cm/yr, respectively). After adjusting for BMI, the mean annual increase in waist circumference for white females was significantly higher than for black females (0.08 cm/yr vs. -0.07 cm/yr, respectively). These relationships remained significant after adjusting for age at menarche.

**Conclusions:**

Black females had significantly steeper increases in waist circumference over adolescence than white females. After adjusting for BMI and age at menarche, however, the annual increase in waist circumference for black females was significantly shallower than for their white peers. These data suggest racial differences in the deposition of fat over the adolescent period.

## Background

In adolescents, central adiposity is related to chronic disease risk [1-3]. The health risks associated with central adiposity are independent of total adiposity [4,5]. Racial differences in the intra-individual distribution of fat tissue are apparent early in life. In a study of black and white females age 7-10 y matched for body weight and maturation, black females had less subcutaneous and visceral adipose tissue [6]. Some studies suggest that the strength of the relationship between central adiposity and cardiovascular disease risk differs by race, although other studies have not supported this notion [7-9].

Distributions for waist circumference, waist-to-hip ratio, and waist-to-height ratio have been constructed from cross-sectional surveys of pediatric populations from a wide range of geographic areas [10-16]. In general, these studies show that waist circumference increases with increasing age during childhood and adolescence, with males having higher values than their female peers. There has been little research on how growth in central adiposity differs by race in a longitudinal dataset. It is also unknown whether differences in central adiposity are independent of the age-related racial divergence in total adiposity, and independent of differences in maturational timing between black and white adolescents. A characterization of individual trajectories of waist circumference, utilizing longitudinal data, would allow the examination of changes in central adiposity over adolescence.

We hypothesized that the observed differences in body composition between black and white adult women are present in adolescence. We tested the hypothesis that differences in the age-related growth in central adiposity between black and white females, as measured by waist circumference, are evident during the adolescent period.

## Methods

### Data

This study used publicly available data from the National Heart, Lung, and Blood Institute NHLBI Growth and Health Study (NGHS), which was established to investigate how dietary patterns, physical activity levels, and psychosocial factors are related to the development of obesity in girls. The study has been described previously in detail [17]. Briefly, this multi-center longitudinal study was composed of girls in racially concordant households (race self-declared as black or white, with Hispanic children excluded) from three study sites: the Richmond School district near Berkeley, CA; Cincinnati, OH; and the Washington, D.C. area. Of eligible girls, 78% were enrolled. At each study center, the respective IRB approved the NGHS protocol, and all participants and their parents gave informed consent. We analyzed 9 years of data from 2379 girls (1213 black and 1166 white) enrolled at age 9-10 years in 1987-1988 and followed annually. The public release dataset of NGHS does not include females who were pregnant at the time of their visit or who had been pregnant in the four months previous to their annual visit. Our investigation was approved by the Institutional Review Board at Tufts Medical Center and Tufts University Health Sciences Campus.

### Measurements

All NGHS measurements were taken according to study protocol by certified, trained staff that were annually retrained and were monitored for consistency [17]. At each annual visit, height and weight were measured in duplicate. Height was measured to the nearest 0.1 cm in subjects wearing socks, using custom-made stadiometers, and weight was measured using Health-O-Meter electronic scales, to the nearest 0.1 kg, with subjects in gowns. Minimum above-waist circumference was measured against the skin, in duplicate, annually beginning with NGHS visit 2. BMI was calculated as weight in kilograms divided by the square of height in meters. Study participants were asked annually whether they had started having their menstrual periods; the dataset included self-reported age at which periods started, measured in years to one decimal place. Trained female nurses assessed sexual maturation using pubic hair distribution and areolar development. Age at menarche and modified Tanner staging principles were used to classify subjects into four maturational stages: prepubertal (premenarcheal and at stage 1 in both areolar and pubic hair development), pubertal (premenarcheal and at stage 2 or greater in either areolar or pubic hair development), <2 years post-menarcheal, or >2 years post-menarcheal [18].

### Statistical Analyses

Subjects who had waist circumference measurements at 3 or more annual visits were included in this analysis. Descriptive statistics were calculated for the sample at NGHS visit 2, the first year that waist circumference was measured in NGHS. For cross-sectional data, t-tests and multiple linear regression were used to compare waist circumference between black and white females at each study visit. Multiple linear regression was used to test whether black and white females had different mean waist circumference after adjusting for age at each of 9 study visits.

Individual growth trajectories of waist circumference were constructed for each subject. We examined each trajectory for outliers and investigated whether a linear model was appropriate for age-related increases in waist circumference. Using PROC MIXED in SAS, we modeled linear longitudinal growth in waist circumference. In our basic models, we fit a univariate linear growth model for waist circumference, investigating the annual change in waist circumference for all subjects, with no other variables in the model. Age in months was treated as the continuous "time" variable in our analysis and "subject" was modeled as a random effect.

We tested whether the increases in waist circumference over adolescence differed for black and white females by including race as a binary variable in our mixed models. BMI was included as a time-varying covariate to test whether increases in waist circumference were independent of increases in BMI. Finally, the role of maturational timing was investigated by formulating models that allowed for a change in waist circumference growth at the time of menarche, permitting both a vertical offset ("jump") in waist circumference and a change in the rate of increase in waist circumference after menarche. In exploratory analyses, we fitted models that incorporated maturational stage data for each subject, allowing for offset and change in slope at each increase in maturation stage. We also investigated whether the racial differences in annual WC change differed by study site, by formally testing an interaction in the model. All analyses were conducted in SAS v9.1 (Cary, NC).

## Results

We analyzed data from 2290 subjects (96.3% of total sample) with >3 annual measurements of waist circumference from NGHS study visit 2 (mean age (SD) 11(0.6) years) through visit 10 (19 (0.7) years). Among these subjects, the mean number of valid waist circumference measurements was 7.9, and 54.8% of subjects had data from all 9 of the study visits analyzed herein. Data were available for 18134 subject-visits.

Subject characteristics at NGHS visit 2, stratified by race, are presented in Table 1. Black females were significantly older, taller, heavier, had higher BMI, and were more sexually mature than white females (all p < 0.05). Compared to subjects who were included in the analysis, those who had fewer than 3 waist circumference measurements did not significantly differ in terms of height, weight, or BMI, according to t-tests at visit 2 (data not shown).

**Table 1:** Subject characteristics at NGHS 2^nd ^visit^1^

	White n = 1106	Black n = 1181
Age (SD)	10.97 (0.56)	11.07* (0.57)
Ht, cm (SD)	145.89 (7.61)	149.50* (8.11)
Wt, kg (SD)	40.52 (10.37)	46.17* (13.03)
BMI (SD)	18.84 (3.63)	20.45* (4.69)
Overweight^2 ^(%)	24.8%	36.4%
Obese (%)	9.2%	20.1%*
Waist Circumference (cm) (SD)	63.60 (8.28)	66.69* (9.82)
Pre-pubertal (%)	29.4%	10.9%*
Pubertal (%)	65.2%	70.5%
<2 years post-menarche (%)	5.3%	17.6%
>2 years post-menarche (%)	0.1%	0.9%

^1^Waist circumference was not measured at baseline in the National Growth and Health Study

^2^Overweight and obesity were defined as the 85^th ^and 95^th ^percentile, respectively, relative to the CDC Growth Reference.

*p < 0.05 by independent.samples t-test or X^2 ^test. Differences remain significant after adjusting for age.

**Figure 1:**
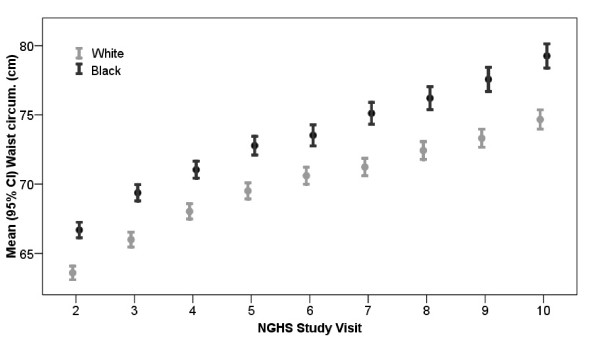
**Waist circumference by study visit**. Cross-sectional comparisons of mean waist circumference and 95% confidence intervals by race and visit

Figure 1A shows that the mean and the variability of waist circumference increased with age. The variability of waist circumference in black females was larger relative to white females. Black females had higher mean waist circumference than white females at each visit, from baseline (66.7 cm vs. 63.7 cm, respectively, means adjusted for age, p < 0.05) through the final visit (79.1 cm vs. 74.8 cm, respectively, adjusted means, p < 0.05, Table 2).

**Table 2:** Cross-sectional comparisons of waist circumference (WC, adjusted means^1^) between black and white females

Visit		Baseline	2	3	4	5	6	7	8	9	10
White	n	n/a^2^	1099	1062	1022	950	826	868	910	941	994
	Mean WC^1^	-	63.7	66.1	68.1	69.6	70.6	71.3	72.5	73.4	74.8
Black	n	n/a	1175	1147	1115	1074	955	963	987	985	1061
	Mean WC	-	66.7*	69.3*	71.0*	72.7*	73.5*	75.1*	76.2*	77.5*	79.1*

^1^Means adjusted to account for small differences in age

^2^Footnote: Waist circumference was not measured at baseline in the NGHS

*p < 0.05 by independent samples t-test. Differences remain significant after adjusting for age.

**Figure 2:**
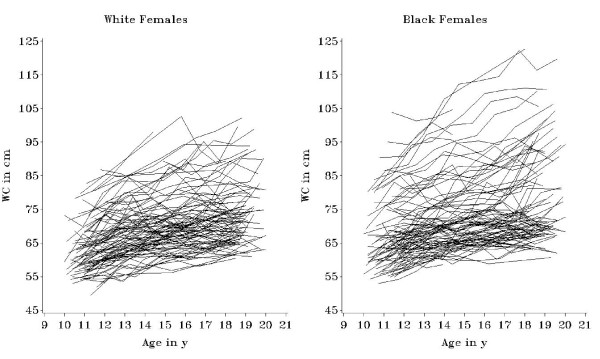
**Individual waist circumference trajectories**. Waist circumference trajectories for a randomly selected subset of 100 black and 100 white NGHS adolescent females

We generated and investigated growth trajectories for each subject. Figure 2A shows waist circumference trajectories for 100 black and 100 white females randomly selected from the sample, with subjects' annual waist circumference data joined with lines. Examination of all growth trajectories showed that variability exists in waist circumference between individual adolescent subjects, in terms of the initial value at study visit 2, the increase over time, and the shape of the individual growth trajectories. Visual examination of trajectories for all subjects also suggested that white females in the NGHS tended to start with a lower waist circumference (at age 10-11), and had a shallower annual increase in waist circumference over adolescence.

**Figure 3:**
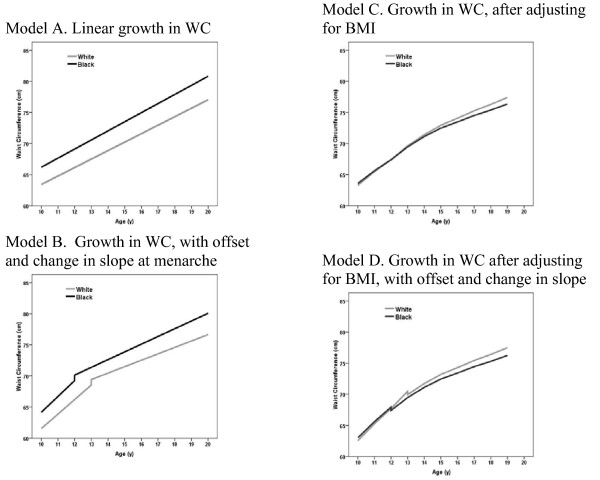
**Prototypical waist circumference trajectories**. Longitudinal waist circumference changes for a prototypical subject with a BMI equal to the mean

Regression models were specified to capture the linear component of change in waist circumference over adolescence for all subjects in the sample. Mean annual increase in waist circumference was significantly higher for black females compared to white females (1.46 cm/yr vs. 1.36 cm/yr, respectively, p = 0.02, Table 3). Figure 3A shows prototypical trajectories for waist circumference growth for the average subject in the sample.

**Table 3:** Racial differences in mean annual increases in waist circumference (WC) in adolescent girls age 11 - 19 y (n = 2378; 18134 subject-visits)

	Model A	Model B	Model C	Model D
Model details (PROC MIXED)	Fixed effect of race	Fixed effect of race, allowing for vertical offset and change in WC slope at menarche	Fixed effect of race, adjusting for annual measures of BMI	Fixed effect of race, adjusting for annual measures of BMI, allowing for vertical offset and change in WC slope at menarche

Annual increase in WC (cm), Whites (SE)	1.36 **(0.07)**	2.33 **(0.08)**	0.08 **(0.02)**	0.62 **(0.03)**

Racial difference in annual increase in WC (Blacks - Whites) (SE)	0.10 **(0.04) **p = 0.024	0.21 **(0.04) **p < 0.0001	-0.15 **(0.01) **p < 0.0001	-0.13 **(0.01) **p < 0.0001

BMI was then added to the model as a time-varying covariate, to test whether age-related racial differences in central adiposity were independent of changes in BMI. The interaction between age and race remained statistically significant after adjusting for BMI, though the relationship was reversed; the mean annual increase in waist circumference for white females was significantly higher than for black females (after controlling for BMI, 0.08 cm/yr vs. -0.07 cm/yr, respectively, p < 0.05). These data suggest that for females growing along a similar BMI trajectory, white females had sharper increases in waist circumference during adolescence compared to black females.

To explore the impact of maturational timing, we expanded our models to permit the regression line to change for each subject at the time of menarche. First, a model was fit that allowed a vertical offset in the regression line at menarche, and an ensuing change in slope. In this model, comparing waist circumference trajectories between black and white females (without adjustment for BMI), the timing of menarche had a significant effect on the vertical offset of the regression line, with menarche associated with a significant increase in waist circumference (p < 0.05). Also in this model, the slope of waist circumference changed at menarche, becoming significantly shallower (p < 0.05). The difference in slopes between black and white females was statistically significant in this model (p < 0.05). When BMI was added to this model, to control for total adiposity, we again saw that black females had significantly lower increases in waist circumference relative to white females, both before and after controlling for the time of menarche (p < 0.05). We also used the detailed maturation stage data to fit models of waist circumference change, adjusting for the four maturational stages. The results were similar as when menarche was added to the model as a dichotomous variable: black females had a smaller annual increase in waist circumference than white females, after adjusting for BMI (data not shown). Finally, in the formal test of the interaction between study site, race and age, we found that the racial differences in waist circumference growth did not significantly differ among the three study sites (data not shown).

## Discussion

In our investigation of the racial differences in central adiposity during adolescence in females we found that, relative to white females, black females have significantly higher waist circumference at each age from 11ï¿½19 y, and have significantly steeper increases in waist circumference over this age range, reflecting their greater adiposity. After adjusting for BMI, however, we found that racial differences persisted but that the annual increase in waist circumference for black females during adolescence was significantly shallower than for white females, suggesting racial differences in central fat deposition over the adolescent period. This observation is consistent with studies on adults, which suggest that black women have less visceral fat than white women when total adiposity is taken into account [19,20]. Studies of younger children report similar findings regarding fat distribution between blacks and whites; white girls have relatively more total and intra-abdominal fat than black girls of similar BMI from age 8 to 13 [21-23]. These differences, which arise after menarche and increase during adolescence, might play a role in the development of cardiovascular disease risk factors over this critical period. Although the clinical significance of the mean difference between black and white females is currently unknown, we would predict that in the subset of females with a change greater than the mean difference, the clinical significance might be important.

Data from adults suggest that central adiposity, independent of total adiposity, is more strongly related to chronic disease risk in white women compared to black women [24,25]. It is not clear whether such differences in disease risk factors are evident in childhood and adolescence. A study of 40 black and white females age 7-10 y found differences by race, with subcutaneous adipose tissue (measured by MRI) positively related to insulin concentrations in black females, but not in whites [6]. There were not significant difference in lipoprotein profiles between the two groups. In contrast, in a cross-sectional study of 61 prepubertal black and white females, when visceral fat was measured by computed tomography (CT), the relationship between central adiposity and cardiovascular risk factors (insulin sensitivity, triglycerides, and HDL cholesterol) did not differ significantly by race after adjusting for total body fat [9]. A similar null finding was reported in a cross-sectional study of 50 black and white overweight adolescents with regard to visceral fat measured by CT and insulin sensitivity, after controlling for total adiposity [8]. These studies, due to their small sample sizes, may have had limited power to detect a significant difference in central adiposity-related disease risk.

In large epidemiological studies of children and adolescents, only anthropometric measures of central adiposity are feasible. Most research using such measures to investigate racial differences in central adiposity in adolescence has been cross-sectional. For example, waist circumference percentiles are available for various populations by combining cross-sectional data from children of different ages [10-15]. In the U.S., the nationally-representative NHANES data show that waist circumference of females during childhood and adolescence differs by race/ethnic group, with white females having the lowest values, followed in increasing order by black females and then Mexican-Americans [13]. If waist circumference is to be used as a screening tool for CVD risk in children and adolescents, it could serve as either an alternative or as an adjunct to BMI [12]. On the basis of the data presented, whether waist circumference cut-offs should be race-specific is worth consideration.

A strength of our analytic approach is the ability to use the NGHS cohort to move beyond cross-sectional investigations of the racial differences in central adiposity to investigate how key factors like race and maturational timing affect the changes of central adiposity in adolescent females. The NGHS permits the true longitudinal analysis of a large sample of adolescents followed annually, starting at age 10-11 y and followed over the entire adolescent period. Also, this study benefits from a high follow-up rate (89% at the final visit), which strengthens the internal validity of our findings. The use of longitudinal models allows us to take advantage of the repeated measures of the subjects of the NGHS, to model change over time. Furthermore, the rich nature of these data allowed us to statistically control for variables of interest. For example, we were able to adjust for BMI, measured at each annual visit, as a time-varying covariate. Also, we were able to assess the impact of the individual timing of maturation, which is important given the earlier maturation of black adolescents, including those enrolled in NGHS [26].

A limitation of our approach is the use of anthropometric measures for central adiposity, and the use of BMI as an estimate of total adiposity. Ideally, to estimate the role of visceral adipose tissue independent of total body fat, criterion measures such as CT and DXA would be employed. Use of these measures is preferable compared to BMI; because BMI only considers weight, it does not distinguish other potential differences in body composition between the races, such as the higher bone density of blacks relative to whites [27]. Such "gold standard" measures, however, are not feasible in a large epidemiological study such as NGHS. The use of waist circumference and BMI permits large sample sizes with sufficient statistical power to detect important differences. Although these anthropometric measures can introduce measurement error, such error is likely to be random and thus would not bias the observed results. Also, the visceral adipose tissue depot, which is quite small during adolescence, might not be the most important aspect in the relationship between central adiposity and cardiovascular disease risk factors [28]. Therefore, a measure such as waist circumference might be more appropriate, inasmuch as it captures subcutaneous abdominal fat as well, which has been implicated as having a role in insulin resistance in children with small amounts of visceral fat [29].

Another limitation of this study is the generalizability of our findings beyond the NGHS sample. This large cohort of black and white females, enrolled in the late 1980s from three different regions of the U.S., from diverse backgrounds, was not designed to be representative of the U.S. population or any particular subset. Therefore, the descriptive statistics characterizing our sample may not be comparable to the contemporary U.S. population during the current era of high rates of pediatric obesity, for example. However, U.S. data from NHANES III, collected from 1988-1994 when 25.6% of girls age 9-11 y were overweight (BMI percentile above the 85^th ^CDC percentile) and 11.0% were obese (>95^th ^percentile), suggest that the NGHS cohort had similar rates of overweight and obesity; at baseline, 9-10 y, 33.4% of blacks were overweight and 17.7% were obese, compared to 22.5% of whites who were overweight and 7.9% who were obese [30]. In the final year of the NGHS, the cohort also had obesity rates comparable to the U.S. as a whole. NHANES 1999-2000 reports the prevalence of overweight for females age 12-19 y at 25.4% in whites and 45.4% in blacks; this compares to 22.9% of whites and 43.7% of blacks in the NGHS data analyzed herein. Also, the prevalence of obesity in NHANES 1999-2000 and the final year of NGHS was similar: 12.4% of whites and 26.6% of blacks in NHANES, compared to 10.9% of whites and 24.7% of blacks in NGHS [31]. Although the generalizability of the NGHS is limited, given the data on obesity prevalence and our observation that the effect of race on age-related changes in waist circumference did not significantly differ between the NGHS study sites, it is possible that such a relationship would be seen in other longitudinal datasets.

## Conclusions

In conclusion, black and white females had significantly different patterns of growth in central adiposity during adolescence. Average annual increase in waist circumference was significantly higher for black females compared to white females. However, blacks had smaller annual increases in waist circumference after adjusting for BMI. Although at the individual level, the annual differences may be small, at the population level a shift of this magnitude (~1 cm over adolescence) could impact public health. Future research should thus address the effect of differential growth in waist circumference on clinical outcomes of interest.

## Competing interests

The authors declare that they have no competing interests.

## Authors' contributions

DJT was responsible for the study design, data analysis and writing of the manuscript, under the supervision of AM. GED provided statistical consultation. AHL and SRD provided critical input and advice. All authors contributed manuscript revisions and approved the final version. None of the authors have any conflicts of interest to report. None of the other authors had a conflict of interest to disclose.

## Pre-publication history

The pre-publication history for this paper can be accessed here:

http://www.biomedcentral.com/1471-2431/10/2/prepub
